# Chronic Thoracic Aortic Dissection: How to Treat, When to Intervene

**DOI:** 10.3390/life12101511

**Published:** 2022-09-28

**Authors:** Panteleimon E. Papakonstantinou, Dimitra Benia, Dimitrios Polyzos, Konstantinos Papakonstantinou, Filippos-Paschalis Rorris, Fotios Toulgaridis, Konstantinos Manousiadis, Sotirios Xydonas, Antonios Sideris

**Affiliations:** 1 Second Cardiology Department, Evangelismos Hospital, 106 76 Athens, Greece; 2 First Cardiology Department, Evangelismos Hospital, 106 76 Athens, Greece; 3 Throracic and Cardiovascular Surgery Department, Evangelismos Hospital, 106 76 Athens, Greece

**Keywords:** aortic dissection, chronic aortic dissection, thoracic aortic aneurysm, TEVAR, frozen elephant trunk, aortic disease

## Abstract

Thoracic aortic dissection (AD) is associated with increased morbidity and mortality. Acute aortic syndrome is the first presentation of the disease in most cases. While acute AD management follows concrete guidelines because of its urgent and life-threatening nature, chronic AD is usually overlooked, although it concerns a wide spectrum of patients surviving an acute event. Acute AD survivors ultimately enter a chronic aortic disease course. Patients with chronic thoracic AD (CTAD) require lifelong surveillance and a proportion of them may present with symptoms and late complications demanding further surgical or endovascular treatment. However, the available data concerning the management of CTAD is sparse in the literature. The management of patients with CTAD is challenging as far as determining the best medical therapy and deciding on intervention are concerned. Until recently, there were no guidelines or recommendations for imaging surveillance in patients with chronic AD. The diagnostic methods for imaging aortic diseases have been improved, while the data on new endovascular and surgical approaches has increased significantly. In this review, we summarize the current evidence in the diagnosis and management of CTAD and the latest recommendations for the surgical/endovascular aortic repair of CTAD.

## 1. Introduction

Aortic dissection (AD) is a fatal disease [1]. The Global Burden Disease 2010 study showed that the overall global death rate from aortic dissection (AD) and aortic aneurysms increased from 2.49 per 100,000 to 2.78 per 100,000 inhabitants between 1990 and 2010 [2,3]. As in other arterial diseases, an aortic disease may have an acute presentation or it may be diagnosed after a long subclinical phase. There are two clinical patterns in patients with AD [1]: patients with initially acute AD entering the chronic phase of the disease, and patients in whom the first diagnosis of chronic AD is made. Patients with chronic thoracic AD (CTAD) also include those previously operated for type A AD, with persisting dissection of the descending aorta. Patients with newly diagnosed chronic AD are often asymptomatic, and the exact timing of dissection is often difficult to determine [1].

Acute aortic syndrome is the first presentation of the disease in most cases [1]. In fact, acute AD survivors ultimately enter a chronic aortic disease course. The management of patients with CTAD is challenging as far as determining the best medical therapy and deciding on intervention are concerned. Although thoracic AD is an important clinical entity with high morbidity and mortality, even in the chronic phase, the available data concerning the management of CTAD is sparse in the literature. The 2001 ESC Task Force on the diagnosis and management of AD was one of the first documents in the world relating to disease of the aorta [4]. However, the 2014 ESC Guidelines on the diagnosis and treatment of aortic diseases was the last ESC Task Force on AD [1]. Since that time, the diagnostic methods for the imaging of aortic diseases have been improved, while the data on new endovascular and surgical approaches has increased significantly. Until recently, there were no guidelines or recommendations for imaging surveillance in patients with chronic aortic dissection. In a recent scientific statement from the American Heart Association, imaging and measurement techniques for patients with chronic aortic dissection are presented, while the need for standardized measurements and reporting for lifelong surveillance are clarified [5].

In this review, we present the current evidence regarding the diagnosis and management of CTAD and the latest recommendations for the surgical/endovascular aortic repair of CTAD.

## 2. Definitions and Classification

AD is a condition caused by an intimal tear that propagates antegradely or retrogradely and separates the aortic layers (intima from media), creating a false lumen that expands at the expense of the true lumen, thereby jeopardizing the integrity of the aorta and its branches. It can be the result of a vasa vasorum rupture with subsequent hemorrhage within the aortic wall. AD is the catastrophic result of an aneurysmal disease. The most common risk factors associated with aortic dissection are older age, uncontrolled hypertension, smoking, cocaine abuse, genetic aortic syndromes (Marfan, Ehlers-Danlos, Loeys-Dietz), congenital diseases like bicuspid aortic valve, coarctation of the aorta, Turner syndrome, chest trauma, prior cardiac surgery and procedural instrumentation, and inflammation-vasculitides (giant cell arteritis, syphilis, Takayasu’s arteritis) [6,7]. Pregnancy is also strongly associated with aortic dissection [6].

The DeBakey classification, focusing on ascending or descending involvement in the dissection process and at the initial entry tear, divides AD into three categories: I-involving ascending, descending aorta and aortic arch, II-confined to the ascending aorta, III-confined to the descending aorta [8,9]. The Stanford classification, taking into account only the extent of the dissection process irrespective of the initial entry point, divides AD into two groups: A: Any ascending aortic dissection; B: Any dissection that does not involve the ascending aorta [8]. However, in neither of the above classifications dissections limited to the aortic arch or dissections arising distal to the left subclavian artery but not involving the ascending aorta are adequately represented (10% of all dissections) [2]. Lansman et al. [10] suggested a classification that takes into account the origin and the propagation of the dissection. Von Segerssen et al. [11] introduced the term non-A-non-B for dissections. The ESC 2014 aortic guidelines make no comment regarding arch dissection, whereas the 2010 AHA guidelines suggest managing dissections of the descending aorta with entry tear within the arch as proximal type B dissections. Dissections involving the arch are associated with a greater need for urgent or late open aortic surgery or aortic interventions and aortic events after a mean follow-up.

The development of modern imaging techniques, able to detect subtle pathological signs, and of various treatment strategies, gave the chance to further refine the anatomic classification scheme of AD [12]. The new classification system, TEM, takes into account the dissection’s extension (T-Type of dissection: A, B, non A-non B), the primary entry location (E-0: no entry tear visible, 1: entry tear within the aortic root or ascending aorta, 2: entry tear within the aortic arch, 3: entry tear distal to the aortic arch), and end-organ malperfusion (M-0: no malperfusion present, 1: coronary malperfusion, 2: malperfusion of the supra-aortic branches, and 3: malperfusion of the spinal cord, viscerals, renals, lower extremities, or a combination of these). (+) is added when a combination of radiographic and clinical malperfusion is present [13,14]. Another classification system is the SVS/STS scheme. Based on the place of entry tear, the dissection is classified as A or B. Then, the aorta is divided into 12 aliquot zones (0–11) from the proximal to the distal part, so that the distal extent of the dissection is designated by zone. For type A dissection, the entry tear is located in zone 0. In type B dissections, the entry tear originates in zone 1 or beyond. Two subscripts further characterize type B dissections, letter P describes the proximal zone, and letter D the distal zone of the involved aorta. For instance, B1,9 dissection describes a proximal involvement in zone 1, distal extension in zone 9 and the entry tear anywhere between 1–9. If the entry point is not identified, the dissection is characterized as I. The vast majority of chronic dissections are patients with a history of dissection with or without repair [15]. This group is at risk of subsequent aortic events (acute on chronic dissection) and the way they can be classified can be really complicated as it is necessary to report previous pathology, type of repair and current residual anatomy [15]. The new classifications supply specialists with important information predictive of outcome (entry tear location, malperfusion) and can further guide the therapeutic approach. In daily practice, however, the Stanford classification supplemented with the term non-A-non-B suffices for other healthcare professionals [1,6]. The aforementioned classifications were developed to assist clinicians in the management of AD. Available classification systems of aortic dissections are summarized in Table 1.

As regards timing, AD can be described as acute, subacute, and chronic (<14 days, 15–90 days, >90 days from symptoms onset, respectively) [1].

While acute AD management follows concrete guidelines because of its urgent and life-threatening nature, chronic AD is usually overlooked, although it concerns a wide spectrum of patients surviving an acute event. Patients with chronic AD do not share uniform features, as their profile is shaped based on the initial nature of AD, the different survival rates among the subtypes (type A vs. type B), and the clinical course (complicated vs. uncomplicated for type B) which dictates different optimal treatment method (conservative, endovascular, or surgical treatment).

## 3. Presentation, Diagnosis and Follow-Up

The largest group of patients with chronic AD (~60%) are those who survived the acute phase of type A AD and were treated surgically, but there is a residual false lumen distal to the surgical repair [5]. A smaller proportion of patients is considered totally cured when the dissected segment is totally resected without the persistence of false lumen [5]. An even more scarce group of patients, considering the life-threatening nature of the disease, are those with type A AD not diagnosed in the acute phase due to absent or atypical symptoms, who survived and entered a chronic phase [5]. The second highest frequency group of patients with chronic AD is represented by those initially diagnosed with type B AD who followed an uncomplicated course and were treated conservatively (~25%) [5]. The third fraction of patients with chronic AD (~15%) consists of patients with complicated type B AD who underwent endovascular or surgical treatment and survived [5]. The term complicated is used to describe recurrence of pain and failure to control blood pressure, early aortic expansion, malperfusion, and signs of rupture like haemothorax, increasing mediastinal and periaortic haematoma, and dictates a more aggressive approach (endovascular or surgical) compared to the uncomplicated type B AD, which can be managed conservatively [5]. Additional features associated with a worse prognosis are the false lumen diameter, the site of intimal tear, and retrograde dissection into the aortic arch. Finally, acute aortic syndromes apart from AD include intramural haematoma and penetrating aortic ulcer, which could also progress into a chronic phase requiring similar management to chronic AD [5].

Consequently, patients with chronic AD can be divided into two categories [1]. The first consists of patients who have received medical, endovascular, or surgical treatment for an acute AD, survived 90 days after the initial event entering a chronic phase, while the second consists of patients with a diagnosis of chronic AD for the first time [8]. These patients require lifelong surveillance with imaging. Imaging studies should evaluate the entire aorta, as the dissected part may remain stable, progress, become aneurysmal, or heal. The report should refer to the result of the initial treatment approach, to any difference from baseline or subsequent scans, and to any new pathologies (presence of aneurysms, malperfusion, or entry flow relative to andografts). The follow-up intervals should be individualized. After the predischarge baseline scan, surgically repaired AD should be followed up at 6 months, 12 months, and annually, thereafter. For patients that remain stable over time, longer follow-up intervals are recommended. Medically managed type B AD should be scanned 1–3 months after the initial event, followed by 6-month, 12-month and annual follow-up. Endovascular treatment requires scanning 30 days after the procedure (early complications) and subsequent monitoring at 6 and 12 months. Annual follow-up is necessary, even in stable patients. Follow-up intervals should be individualized in the case of abnormal findings requiring closer monitoring [5]. Due to the chronic nature of the disease and the need for many serial measurements, a standardized protocol on how to perform, measure, compare, and report is very important [5]. Patients with a diagnosis of CTAD for the first time are quite rare, usually totally asymptomatic, and only incidentally detected while undergoing scanning for other purposes.

When symptoms appear, they are usually associated with aortic expansion and subsequent aneurysm formation or with urgent late complications [1,5]. Aneurysms are detected in 34–38% of patients with chronic type B AD and in up to 49% of patients operated for type A AD with residual chronic AD in the distal aorta [5]. Possible symptoms are chest, abdominal or back pain, shortness of breath, cough, hoarseness, and dysphagia [1,16].

Malperfusion can manifest as lower limb ischaemia or mesenteric ischaemia, causing claudication or abdominal pain, or as deteriorating renal function [1,5]. Patients with chronic AD remain at risk of new intimal tears in the remaining aorta [5]. Retrograde type A AD is present in 6.9% of patients with chronic type B AD treated with TEVAR, in 4.3% treated with surgery, and in 3.6% treated medically [5]. It is more common in patients with Marfan syndrome [5]. The most detrimental expression of chronic AD is late aortic rupture, seen in approximately 3–18% of patients [5]. Aortic dissection and rupture both cause acute chest pain [1,5]. Obtaining a careful medical history directed to symptom onset is necessary in patients with an incidental finding of AD in an attempt to reveal the exact timing of dissection. The clinical examination focuses mainly on the cardiovascular and neurologic status. Blood pressure, murmurs, and peripheral pulses must be assessed. Electrocardiography may show ischaemic changes or hypertrophy, while a chest x-ray may reveal a widened mediastinum or a prominent aortic knob [1,17]. Non- invasive imaging techniques continue to be the gold standard for diagnosing both acute and chronic AD [1,6]. In chronic AD, aortic dimensions, intimal flap motion and thickness, false lumen patency, and aneurysm formation should be evaluated [5]. In symptomatic patients, findings compatible with rupture (pleural effusions, mediastinal haematoma) may be found [1]. Advantages and limitations of each diagnostic modality are described in Table 2.

The follow-up of patients with chronic AD is mainly based on symptoms and on the findings of the imaging modalities. Aortic expansion > 60 mm, annual aortic growth rate > 10 mm/year, persistent patency of the false lumen (thrombosed lumen has a beneficial impact on aortic enlargement and long-term survival), recurrent pain, and signs of end organ ischaemia are indicative of instability, so TEVAR or surgery is recommended [1,5,18]. In uncomplicated chronic AD, annual clinical and imaging evaluation is recommended, irrespective of the treatment method previously applied [1]. Computed tomography angiography is the imaging method of choice for the follow up of patients with chronic AD.

## 4. Medical Treatment

### 4.1. Blood Pressure Control-Antihypertensive Medication

The available evidence on medical management of chronic aortic dissection is lacking. Current guidelines from the European Society of Cardiology [1], American College of Cardiology/American Heart Association [19] and Japanese Circulation Society [20] reaffirm the lack of evidence and suggest adequate blood pressure (BP) control with recommended values lower than 130/80 mmHg and prophylactic long-term therapy with beta-blockers as a potential treatment option. The goals for medically managed patients with chronic type B aortic dissection are generally the prevention of associated complications, such as progression of the dissection, malperfusion, aneurysmal degeneration, and rupture [21].

Beta-blockers effectively lower heart rate and blood pressure (BP) and reduce the peak ejection rate of the left ventricle. They also decrease left ventricular dP/dt and reduce shear stress on the aortic wall [22], presenting their potential beneficial role in chronic aortic dissection.

Genoni et al. compared the treatment with beta-blockers vs. other antihypertensive drug classes in patients with aortic dissection type B during a follow up period of 4.2 years. They showed that the rate of increase in aortic diameter was lower in the beta blocker group compared to the other treatment group (12% vs. 40%, *p*-value = 0.002). Furthermore, a lower percentage of patients that received treatment with beta-blockers needed surgery due to dissection-related events during follow up (18% vs. 55%, *p*-value = 0.002), while the duration of hospitalizations related to aortic dissection was lower for patients on beta-blockers (*p*-value = 0.001) [23].

A recently published retrospective analysis compared Angiotensin-Converting Enzyme (ACE) inhibitors/Angiotensin II Receptor Blockers (ARBs) and beta-blockers with other antihypertensive agents as long-term medication in patients with aortic dissection over a span of up to 12 years. Compared to the control group, beta-blockers (hazard ratio [HR], 0.82; 95% confidence interval [CI], 0.73–0.91) and ACE inhibitors/ARBs (HR, 0.79; 95% CI, 0.71–0.89) were associated with lower all-cause mortality. Furthermore, the risk of all-cause hospital readmission was lower for the patients that received ACE inhibitors/ARBs (HR, 0.92; 95% CI, 0.84–0.997) and beta-blockers (HR, 0.87; 95% CI, 0.81–0.94) compared to the patients that received the other drug classes. Moreover, there were no statistically significant differences in other endpoints including death due to aortic aneurysm/dissection, major adverse cardiac and cerebrovascular events, and new-onset dialysis, between the two medication groups [24]. This study’s findings suggest that ACE inhibitors and ARBs could be used instead of beta-blockers to treat patients with chronic aortic dissection. More evidence from randomized clinical trials is required to assess the benefits and harms of beta-blockers and ACE inhibitors/ARBs as first line treatment in patients with chronic aortic dissection.

Suzuki et al. investigated the effects of antihypertensive medication on mortality in a registry of patients with aortic dissection for a follow-up time of ≤5 years. The analysis showed that use of calcium channel blockers (CCBs) was associated with improved survival in medically treated patients with type B aortic dissection in univariate analysis (*p*-value = 0.03), as well as in multivariate analysis (odds ratio, 0.55; 95% CI, 0.35–0.88) [25]. For other antihypertensive medications such as diuretics, alpha blockers, centrally acting drugs, and others, there is no available data and recommendations in the setting of chronic aortic dissection. These agents would be beneficial for their antihypertensive effects, but clinical trials are needed in order to determine the role of these agents in patients with chronic aortic dissection.

In summary, pharmacological management of chronic aortic dissection is still determined by personal experience, expert opinion, and outdated observation studies, while contemporary randomized controlled trials are lacking. Beta-blockers are considered as the cornerstone medication due to their properties and mechanisms of action; however, updating clinical practice guidelines based on recent and valid evidence are necessary.

### 4.2. Lipid-Lowering Agents

Since their initiation in clinical practice in the late 1980s, statins have become widely used as the primary and secondary prevention therapy in patients with cardiovascular risk factors and cardiovascular disease [26]. In addition to the lipid-lowering properties, their pleiotropic actions, including anti-inflammatory and anti-atherosclerotic effects, established statins as the cornerstone therapy for these patients [27]. Statins, in particular, have been shown in studies to reduce endothelial inflammation, improve atherosclerotic plaque regression, and slow collagen degradation by stabilizing imbalances in matrix metalloproteinases (MMPs) and their inhibitors [28].

Recent published data points out the beneficial role of statin therapy in the pharmaceutical management of aortic diseases. Studies suggest that statins are linked with fewer complications in patients with aortic aneurysm [29,30]. However, the available data regarding the role of statins in chronic aortic dissection is still scarce. In a prospective randomized trial, Masaki et al. showed that the addition of pitavastatin to standard therapy with antihypertensive agents in patients with uncomplicated type B aortic dissection led to lower rate of increase in the diameter of the aortic arch during the 1-year follow-up period [31]. Autophagy is an essential catabolic pathway which is considered as a cytoprotective mechanism that controls the function of endothelium, while recent studies demonstrate the important role of autophagy in the pathogenesis of aortic dissection [32]. Peng et al. identified atorvastatin as a potential autophagy-inducing agent, through the suppression of the phosphorylation process of the enzyme mTOR. Atorvastatin modified the expression of enzymes such as LC3-B and p62 that participate in autophagy, leading to a decreased activation of the inflammasome NLRP3 and to reduced inflammation [33].

### 4.3. Diabetes and Antidiabetic Medication

Data derived from systematic reviews and meta-analyses support that the prevalence of aortic disease, including aortic dissection, is lower in patients with diabetes, while diabetes also acts in a protective way for aortic dissection. Furthermore, one possible mechanism for the paradox phenomenon suggests that hyperglycemia alters factors involved in aortic wall inflammation, such as collagen and elastic fiber synthesis, as well as MMP activation; however, the underlying mechanisms for these negative correlations remain unknown [34,35].

A few studies have suggested the aortoprotective effect of antidiabetic agents on abdominal aortic aneurysm, while incretin mimetic drugs such as glucagon-like peptide 1 (GLP-1) receptor agonists and sodium-glucose cotransporter 2 (SGLT2) have also been investigated for their effects on aortic disease in a few animal studies [36,37]. On the other hand, evidence regarding the role of antidiabetic medication in chronic aortic dissection remains poorly characterized. Wang et al. demonstrated that sitagliptin, a dipeptidyl peptidase-4 (DPP-4) inhibitor, enhanced the autophagy process and attenuated endothelial dysfunction in obese diabetic rats [38]. In summary, the impact of antidiabetic treatments on chronic aortic dissection development and progression is still unknown, and our knowledge is based on circumstantial evidence. The effect of antidiabetic medication on pathophysiological pathways and mechanisms involved in chronic aortic dissection is only partly determined, therefore, further research and clinical trials are needed to investigate the role of antidiabetic agents as a promising therapeutic strategy in patients with chronic aortic dissection.

A summary of the studies regarding the role of medical treatment in chronic aortic dissection is presented in Table 3.

## 5. Interventional Treatment

### 5.1. Chronic Aortic Dissection

Chronic aortic dissection refers to, in most cases, patients with residual dissection in the downstream aorta, either after Stanford type A or B dissections. Incidentally found chronic aortic dissections are quite rare in everyday clinical practice and comprise a group of patients that present with atypical or even absent symptoms at the acute dissection event that are untreated and that progress to the chronic phase of the disease. Usually, CTAD is an incidental finding in chest x-rays, where mediastinal widening or an enlarged aortic knob is encountered [39]. Symptoms of aortic enlargement may include the involvement of adjacent structures (hoarseness or recurring chest pain), malperfusion syndrome (such as vision changes, syncope, extremity pain), or valvular heart disease due to aortic insufficiency [40]. Interestingly, bicuspid aortic valves are more frequently seen in CTAD patients [41]. Additionally, chronic histologic changes may occur, rendering CTAD a distinct pathologic process from that of aortic aneurysms. Currently, there are no formal guidelines that specifically address CTAD, and treatment relies on generally accepted diameter thresholds for aortic aneurysms [1], although lower thresholds may be considered [42].

Multiple entry tears in the acute dissection event, inadequate proximal repair, and distal anastomosis new entry tears (DANE) prevent false lumen thrombosis and thus facilitate aortic enlargement in the long term. Reoperations for residual aortic dissections are indicated when the aortic diameter has reached the guideline threshold, i.e., 5.0 or 5.5 cm. The surgical management of such patients must be individualized after careful and thorough study of the CT scan.

Concerning chronic type B aortic dissections, almost 25–50% of patients with acute type B aortic dissection who were treated conservatively will require future treatment (surgical or endovascular) due to aneurysmal dilation of the chronically dissected thoracic aorta [43]. Chronic uncomplicated type B aortic dissection is initially treated with medical therapy along with clinical and imaging follow-up [44]. However, complicated type B aortic dissections (CBAD), meaning those with rapid thoracic aortic enlargement (>10 mm/year), false lumen aneurysms (with a total aortic diameter of > 60 mm), recurrent pain, or malperfusion syndrome [1], require treatment, either surgical or endovascular. Aneurysmal dilatation and rapid aortic growth are the most frequent indications for treatment [43].

### 5.2. Surgical Interventions

The classic two-stage Elephant Trunk (ET) operation followed by subsequent replacement of the thoracoabdominal aorta was considered the gold standard for the management of aortic dissections of the thoracoabdominal aorta involving the aortic arch. This technique is still being used in the United States, where the hybrid prostheses for the Frozen Elephant Trunk (FET) operation are not commercially available. Only recently has the Thoraflex hybrid system received approval from the Food and Drug Administration. The classic approach is associated with high interval mortality, i.e., the period between the two operations. However, the technique is the preferred surgical treatment of choice in young patients and in patients with connective tissue disorders.

FET is indicated for post-dissection aneurysmal formation after type A repair, as well as for treatment of type B aortic dissections, both acute and chronic. The following parameters should be considered: (a) The location of the segment with the greatest diameter, because the closer it is, the more likely the stent graft will achieve satisfactory results; (b) the true lumen size, because if it is too small, the risk of pseudocoarctation after FET is increased. However, as with thoracic endovascular aortic repair (TEVAR), true lumen expansion may be inadequate, the false lumen may still be perfused through communications between the two lumina, and the rigid dissecting membrane may rupture due to increased force induced by the stent–graft at its distal end [45]. FET is also indicated in the post-dissection aneurysm formation of the aorta after chronic type B aortic dissection. Indeed, it is a viable option, when the aortic pathology includes the arch (aortic arch aneurysm) when the arch angulation is steep, and when there is no landing zone for TEVAR in select patients. In particular, total aortic arch rerouting has been associated with retrograde type A aortic dissection after TEVAR. In such cases, FET is the preferred treatment modality [46]. Usage of FET in chronic aortic dissection where visceral and renal vessels are solely perfused by the false lumen is possible but should be carefully evaluated in advance. The assurance of patent lumina communications seems mandatory to avoid malperfusion.

Open surgery has traditionally been the cornerstone of treatment of chronic type B aortic dissection and has been the only option available for decades. The principal aim of the surgical treatment is the excision of the primary tear and all thoracic/abdominal diseased aortic tissue using a synthetic Dacron graft. Thus, peripheral and visceral perfusions are restored. The surgical approach includes the institution of full cardiopulmonary bypass and deep hypothermia, with or without circulatory arrest, along with neurologic monitoring and certain protective measures, such as left heart bypass with distal aortic perfusion, cerebrospinal fluid drainage, and reimplantation of critical intercostals and/or lumbar arteries between T8 and L2.

The “distal first” open surgical repair has been very well described by Estrera et al. [47]. Ιn summary, after exposure of the aorta, this includes the institution of a left heart bypass with distal perfusion of the aorta, opening the distal aorta and creating a fenestration in the dissecting membrane to perfuse both true and false lumina distally. After completion of the distal anastomosis, a clamp is placed proximally, and the aorta is opened, and identification of patent intercostals is carried out. The proximal anastomosis is the completed, and carefully selected intercostals (through proper neurophysiologic monitoring) are re-implanted to the graft using another side graft, if necessary.

In the past, high mortality rates and complications were quite frequent [48]. However, with the advent of new techniques, these rates have diminished, but still remain significant: mortality rates of <10% in elective cases; renal failure rates of 8.1%; reintervention for bleeding of 8.1% and spinal cord ischemia of 4.9% [47,48,49].

Total arch replacement with the FET technique is probably the single most important advancement in aortic surgery in the last 20 years and has been increasingly gaining ground in the management of such patients. Since its inception at the beginning of the millennium, the FET operation has dramatically replaced the previous classical elephant trunk with subsequent thoracoabdominal aortic procedures. Although the FET operations have been used for almost 20 years in European and further Eastern countries, the hybrid prostheses were not available in the United States until only recently.

### 5.3. Endovascular Interventions

#### 5.3.1. Chronic Type A Dissection and Residual Aortic Dissection

The endovascular approach of the ascending aorta and aortic arch in chronic aortic dissections is usually reserved for those patients at prohibitive risk for open surgery, or those who refuse surgery. However, its widespread application has been halted by the lack of ascending aorta-specific grafts for aneurysm or dissection, thus leading to off-label use of grafts and custom-made devices. A Computed Tomography (CTA) is used for preoperative evaluation and candidacy for endovascular treatment. Anatomical requirements include a homogenous diameter in the proximal landing zone (maximum sinotubular junction diameter of 38 mm), no sharp angles or calcification between the inner and outer curvature of the aorta, and 20 mm of landing zone both proximally and distally from the aortic pathology treated for adequate sealing. These requirements are seldom fulfilled with the existing devices [50], thus leading to adverse events such as retrograde aortic dissection, endoleaks, and device migration. Proper graft sizing is very important for procedural success [51]. Other pitfalls include the inadvertent obstruction of coronary ostia and the difficulty of crossing a previously implanted mechanical valve. Data from case reports and case series seem encouraging, although most of them apply to acute dissections [50,51]. Additionally, newer techniques may be applied in the future, such as a modified version of the Endo-Bentall, in which a covered stent may be used after the coronary arteries to the ascending aorta to obtain an adequate landing zone. Nevertheless, endovascular treatment of chronic type A aortic dissection is still under investigation and future developments will broaden our treatment options for this disease.

#### 5.3.2. Chronic Type B Aortic Dissection

The minimally invasive nature of endovascular treatment has gradually gained many supporters. During TEVAR, confirmation of at least 2.5 cm proximal and distal landing zone length is mandatory for success. In addition, proximal landing zone extension using surgical techniques prior to TEVAR may be used (left common carotid to left subclavial artery bypass, or both vessels bypassed to the brachiocephalic artery). In order to create a sufficient distal landing zone, endovascular modalities include fenestrated and branched endografts when anatomically suitable. Preoperative evaluation includes assessment of the coronary status with echocardiography and a CTA, which provides affirmation of the feasibility of the endovascular approach. It should extend from the supra-aortic vessels down to the level of the common femoral arteries. The circle of Willis should be assessed if the stent graft is to be deployed in Ishimaru zones 0, 1, or 2. Based on current devices, a minimum diameter of 6 mm of the common femoral arteries is required for the delivery sheaths to be introduced without complications.

Due to diminished aortic plasticity over time in chronic dissection, obtaining an adequate distal seal may be a challenge, since the false lumen may continue to be perfused and new entry tear may emerge because of the graft tension applied to the vessel wall. Concerning distal stent graft-induced new entries (dSINE), due to the rigidity of the dissecting membrane, the aorta may be less vulnerable to this complication over time [52].Concerning false lumen perfusion, several techniques have been used in the radiology suite to address this issue, with varying degrees of success.

The PETTICOAT concept (Provisional Extension To Induce Complete Attachment Technique) has mostly been applied to acute aortic syndromes, though it may be used in chronic dissections as well [53]. It combines the use of thoracic covered stents along with bare metal stents, with the aim of closing the entry tear with the former and expanding the true lumen with the latter. Indeed, this technique helps decrease the pressure in the false lumen (and ultimately leads to false lumen thrombosis) and increases the covered stent graft radial force. Thus, dynamic obstruction of branch vessels (if existent) is resolved.

The STABILISE technique (“Stent assisted balloon induced intimal disruption and relamination in aortic dissection repair”) has been implemented as an adjunct to the PETTICOAT concept. After the deployment of the stents, staged ballooning of the grafts is carried out, with the purpose of immediately repositioning the dissection membrane to the outer aortic layers through obliteration of the false lumen [54].

Concerning false lumen embolization, the “Candy-Plug” technique entails the use of a “candy-shaped” graft to the false lumen, thus limiting persistent false lumen backflow [55]. One of the pitfalls of this technique is true lumen narrowing due to expansion of the “candy-plug” in the false lumen and the possibility of flap or vessel wall injury due to shear stress induced by this special graft [56]. Certain modifications have been proposed [57].

The Knickerbocker technique is another technique of false lumen embolization above the celiac trunk level [58]. It involves the deployment of a double-tapered tubular graft (the Knickerbocker graft) in the true lumen, with the distal landing zone above the celiac artery. This graft has an asymmetric bulbous section that may be expanded with a balloon. The aim is to fenestrate the dissecting membrane after balloon dilation, and thus, approximate the intima to the outer wall of the aorta. Thus, the false lumen is sealed [59].

FEVAR (Fenestrated Endovascular Aortic Repair) and BEVAR (Branched Endovascular Aortic Repair) are two relatively new techniques used for treating arch or thoraco-abdominal aortic pathology [60]. The disadvantages of these grafts include an increased risk of retrograde type A aortic dissection after implantation for arch pathology and the possibility of damaging the aortic wall due to increased wire manipulation. Additionally, a high reintervention rate has been noted due to type IC endoleaks [61]. The use of arch-fenestrated stent grafts in chronic dissections remains very limited.

### 5.4. Comparison of the Different Interventional Approaches for Chronic Throracic Aortic Dissection

Traditionally, chronic type B aortic dissections were treated with open surgery. However, since 1999 and the spawn of the endovascular era, TEVAR has also emerged as a treatment option, given the markedly invasive nature of surgical repair. The rationale for the endovascular treatment of chronic type B aortic dissection stems from the satisfying results of TEVAR documented in the INSTEAD-XL trial [62]. Currently, there are no randomized controlled trials or controlled clinical trials that compare open surgical repair with the endovascular approach for chronic type B aortic dissection [63].

The main purpose of TEVAR in chronic dissection is to eliminate aortic expansion in the chronic phase of a previous acute type A, type B, or non-A-non-B aortic dissection. Favorable results include true lumen expansion, false lumen thrombosis or shrinkage, and stable or diminishing aortic diameter, generally stated as positive aortic remodeling. Although the short-term results of TEVAR may be better than surgical repair, the mid-term outcomes are not as satisfying due to aorta-related complications. These include a persistently patent false lumen leading to aneurysm formation, endoleaks, and increased reintervention rates. False lumen perfusion may lead to a total aortic expansion of 4 mm/year [64].

Ever since 2006, initial results have seemed encouraging [65]. Indeed, at that time, the endovascular approach was deemed to have 2.9–3.4% risk of neurologic complications. 1% risk of paraplegia was considered low at that time compared to 7–36% of surgical repair, and 1.9–2.6% risk of stroke with TEVAR was also considered low. However, these new TEVAR devices were incapable of inducing false lumen thrombosis, with about 12% of patients requiring reintervention during follow-up. In more contemporary series [66], this percentage rises to 20%, with late aneurysmal dilation of the distal aorta being the most frequent cause. New evidence also shows all-cause and aorta-related mortality rates of 1.6% and 0.5%, respectively, stroke rates of 1.1% and spinal cord injury of 0.9% [43].

False lumen thrombosis in the chronic phase is quite variable, ranging from 38–93%, and an increase in false lumen size may be encountered in 15–17% of patients [48]. False lumen thrombosis is critical for long-term success after TEVAR [66].

The chronically dissected aorta sustains certain histological changes over time. These include intimal thickening, decreased flap motion with flap straightening over time, with a thicker flap [52]. Additionally, proximal entry tear sealing does not suffice to treat chronic type B aortic dissection, as distal fenestrations are frequently encountered that continue to perfuse the false lumen over time [49]. False lumen high pressure, along with the more rigid dissection flap, sets boundaries to the effect TEVAR has on positive aortic remodeling [66].

Newer techniques to address the issue of false lumen patency in aortic dissection have not been widely adopted so far and are mainly applied in certain centers and in a small series of patients. Further studies of their use are warranted to elaborate on their application in the radiology suite.

On the other hand, there is the notion that recurrent aortic disease may only be managed with surgical resection of the diseased segment, as it obliterates the possibility of aneurysm formation. This opinion has, indeed, a stable background, as freedom from re-intervention in the surgically resected segment reaches 94% in 20 years [47]. Thus, the durability of surgical repair is undisputed. In addition, the late risk for intervention on an uninvolved aortic segment, e.g., disease progression, was relatively low, with 82% of patients experiencing 20-year freedom from disease progression requiring intervention. However, disease progression after extensive surgical repair (even if the aortic segment is not compromised at the time of the operation) is unpredictable.

Concerning the FET procedure, the stent graft may serve as a secondary landing zone for future aortic intervention, either open or endovascular [46]. It can be clamped during open surgical repair of thoracic or thoracoabdominal aortic pathology and provides a safe landing zone for complementary endovascular extensions.

It is critical that physicians consider the long-term complications of endovascular repair against the risks of open surgery on a case-by-case basis. There is no optimal strategy, but those patients should ideally be treated in centers where both solutions are present, along with expertise and mastery in both endovascular and surgical techniques in a multi-modality team. Thus, the full spectrum of treatment options that will yield the best outcomes will be available.

## 6. Follow-Up after Aortic Intervention

The first follow-up examination after thoracic aortic intervention (TEVAR or surgical thoracic aortic repair) should be performed after 1 month [1]. This is important for the diagnosis of early complications. For TEVAR, these complications include stroke, endoleaks, endograft collapse, vascular access and device delivery injuries, renal failure, aortoesophageal and aortobronchial fistulas, and device failure [67,68]. Additionally, for surgical thoracic aortic repair, the most common complications are anastomotic pseudoaneurysm, graft limb thrombosis, graft infection, and rarely, secondary aortic ruptures. After the first month, surveillance should be performed every 6 months, 12 months, and then yearly [1]. Clinical examination and medical history are important to find signs and symptoms indicative of complications. Symptoms such as chest or back pain, hoarseness, dysphagia, claudication, or abdominal pain are suspicious for complications and further examinations should be performed. Moreover, strict monitoring and control of blood pressure is important for the patient to avoid late complications [67,68].

### 6.1. Imaging Follow-Up Methods

#### 6.1.1. CT Angiography (CTA)

CTA is the gold standard for surveillance imaging after thoracic aortic intervention. The protocol contains a noncontrast unenhanced phase, followed by arterial contrast phase images and finally delayed-phase images. This is helpful to find details such as the density of the aneurysm sac as a result of calcification within the mural thrombus, prior coil material, surgical clips, or a true endoleak. Furthermore, measurements of the aorta are accurate because of the use of 3D multiplanar reformat software [67]. CTA is superior compared to magnetic resonance angiography (MRA) for identifying mechanical graft complications such as kinking, facture, or migration. Additionally, infection of soft tissue, such as inflammation or abscess formation, is more easily recognized on CTA [68]. However, ionizing radiation exposure and intravascular contrast loading are the main reasons for concern about the use of CTA in specific groups of patients, such as young people or patients with severe renal insufficiency [67].

#### 6.1.2. Magnetic Resonance Angiography (MRA)

MRA is used as an alternative to CTA for imaging surveillance after thoracic aortic intervention. The advantages of this technique include avoidance of ionizing radiation and potentially nephrotoxic contrast material [67]. Additionally, MRA has been found equal to CTA in depicting aneurysm size, and superior to CTA in the detection of type II endoleaks [67]. The disadvantages of MRA compared to CTA and other imaging techniques include its higher cost, difficulty with interpretation, and lower availability [67]. Finally, MRA is more useful in patients with nitinol stent grafts because the presence of stainless steel and nickel alloy grafts causes a significant number of artifacts that make the optimal evaluation of MRA difficult [67].

#### 6.1.3. Ultrasound

Ultrasound is an alternative technique for surveillance after EVAR. Its advantages include lack of ionizing radiation, reduced cost, and no use of nephrotoxic contrast. However, ultrasound has some limitations, such as the presence of artifacts in large body habitus patients, interoperator variability, and the difficulty of providing information about stent-graft integrity and complications such as graft migration and kinking [68]. Duplex ultrasound allows the analysis of endoleaks with a similar specificity compared to CTA and the use of contrast-enhanced ultrasound with the use of nontargetedmicrobubbles can offer supplementation to CTA or unenhanced ultrasound in a problem-solving role in difficult cases [68].

#### 6.1.4. Conventional Angiography

Digital subtraction angiography (DSA) is an invasive technique used to monitor patients after aortic intervention, and its use is currently limited to the treatment of endoleaks detected using noninvasive imaging techniques. Furthermore, DSA carries a low but significant risk of complications associated with direct arterial puncture, such as arterial thrombosis, retroperitoneal hemorrhage, pseudoaneurysm, and arteriovenous fistulae [67]. Moreover, CTA and MRA have shown better sensitivity for detecting complications and are preferred over DSA for the surveillance of patients after aortic intervention [67].

## 7. Chronic Thoracic Aortic Aneurysms in Specific Situations and Populations

### 7.1. Sports Activity

Patients that have been submitted to surgical treatment of type-A AD are often restricted from physical exercise due to a lack of knowledge about possible elevation in blood pressure during the time this takes place [69]. This potential danger is also expressed in both European and American guidelines, as physical workload can result in aortic wall shear stress, leading to sudden and unpredictable rises in systemic blood pressure [6,70,71].

Sports activity up to 3–5 metabolic equivalents (METS) per day seems to be a safe and helpful suggestion for both groups of chronic AD, meaning those with type A and type B, as well. Of course, this recommendation needs to be specifically personalized for each case based on the exact characteristics of each individual [72]. Moreover, recent studies suggest that type-A aortic dissection patients have good hemodynamic responses to exercise, indicating that this subgroup of patients matches those seen in cardiovascular disease (CVD) [69]. The latter finding is, to a certain extent, applicable for type B AD patients too.

The European Society of Cardiology (ESC) emphasizes that patients with chronic AD should be discouraged from participating in competitive sports and also in isometric heavy weightlifting. In addition, the previous instruction applies to body-contact sports activities, whereas leisure activities with low static or low dynamic stress are acceptable [70,71].

On the other hand, the American College of Cardiology (ACC)/American Heart Association (AHA), adopts a rather stricter set of recommendations, stating that restrictions should be made not only on competitive sports, but also on lifestyle activities [6,71].

So far, no large-scale randomized control trial (RCT), either multi- or single-centric, has been planned to investigate the hemodynamics, symptoms, metabolic, electrocardiographic, and echocardiographic findings of patients with chronic aortic dissection during physical exercise. There is insufficient data on various types of sports or other physical activities in patients who have had an AD treated surgically, percutaneously, or conservatively.

### 7.2. Pregnancy

Marfan syndrome is the most common underlying cause of AD in pregnant women, followed by pregnancy itself in previously healthy pregnant women with no other predisposing risk factors [73]. In people with heritable thoracic aortic diseases, such as Marfan and Loeys–Dietz syndrome, an aortic root size beyond a certain threshold is a risk factor for the development of AD [74]. Female patients with a history of a previous aortic dissection should be advised against pregnancy, because there is a very high risk of a second AD [75].

## 8. Conclusions

The long-term management of CTAD is mainly based on clinical features. Patients with uncomplicated CTAD are generally medically treated with periodic imaging and clinical surveillance. However, patients who develop complications require intervention. Some patients with clinical characteristics that are predictive of the development of complications may benefit from earlier intervention. TEVAR is the first-line treatment, when anatomically feasible, in patients without connective tissue disorders. For patients who are not candidates for TEVAR, surgical repair provides an alternative option, but is associated with increased perioperative mortality. There are no well-established recommendations for long-term management of CTAD in specific populations. CTAD patients constitute a heterogeneous group of patients who require lifelong surveillance and a proportion of them may present with symptoms and late complications demanding interventional therapy. Consequently, specific guidelines for diagnosis, treatment, and follow-up of CTAD are a necessity. There is a need for large multicenter randomized controlled trials to determine the best medical therapy and the optimum time for surgical/endovascular intervention.

## Figures and Tables

**Table 1:** Anatomic classifications of thoracic aortic dissection.

DeBakey (1965)	**I** Both Ascending and Descending Aorta	**II** Ascending Aorta	**III** Descending Aorta	
Stanford (1970)	**A** involving ascending aorta	**B** ascending aorta is spared		
von Segesser (1994)	**A** involving ascending aorta	**B** ascending aorta is spared	**NON-A-NON-B** limited to the arch/retrograde dissection from descending aorta to the arch not involving ascending aorta	
TEM (2020)	**T** (type) **A** **B** **Non A–non B**	**E** (entry tear) **0** not visible **1** ascendinng aorta **2** arch **3** descending aorta	**M** (malperfusion) **0** absent **1** coronary arteries **2** supra-aortic vessels **3** spinal, visceral, iliac	**(+)** clinical symptoms
				**(−)** no clinical symptoms
STS/SVS (2020)	**T** (type) **A _D_** (entry tear zone 0) **B _PD_** (entry tear ≥ 1) **I _D_** (unidentified entry tear involving zone 0)	**P** (proximal) **0–12**	**D** (distal) **0–12**	

**Table 2:** Imaging methods for the diagnosis and follow-up of thoracic aortic aneurysms.

	Strengths	Weaknesses
Transthoracic Echocardiography (TTE)	-noninvasive -widely available, portable -low cost -chronic AD with concomitant dilatation of aortic root -assessment of aortic regurgitation, pericardial effusion -no contrast agents -no ionizing radiation	-not all segments of the aorta are visualized
Transoesophageal Echocardiography (TOE)	-detect blood flow, false lumen thrombosis and communication between false and true lumen -reserved for haemodynamically unstable patients -no contrast agents -no ionizing radiation	-blind spot in distal ascending aorta-requires sedation
Computed Tomography Angiography (CTA)	-First line investigation -high spatial resolution -short acquisition time -helpful for diagnosis, follow-up -first choice in the context of endovascular aortic stent-grafts, mechanical valves and for endoleaks assessment -able to detect multiple entry tears	-radiation exposure (effective radiation dose from single CT scan 2–20 mSv and 3–60 mSv for 3-phase scan) -contrast agents required
Magnetic Resonance Imaging (MRI)	-no ionizing radiation (important especially for young patients requiring long-term follow up) -high sensitivity, specificity -apart from accurate anatomic information allows for aortic regurgitation assessment and aortic physiology investigation (flow, stiffness, elasticity, shear stress) -intimal flap, blood flow assessment, false lumen thrombosis	-longer scanning time -lower spatial resolution -incompatible with stainless steel implants -higher cost -low availability
Positron Emission Tomography-FDG-PET/CT	-diagnosis, risk stratification, treatment plan -differentiate acute from chronic AD in unclear cases (increased FDG uptake in the AD membrane and adjacent aortic wall in the acute phase compared to the low metabolic activity in stable chronic AD)	-Further validation needed
Aortography	-side branch/coronary artery involvement	-invasive -need for contrast -radiation exposure -risk of further iatrogenic dissection
Intravascular Ultrasonography (IVUS)	-high specificity and sensitivity	-invasive

**Table 3:** Summary of studies regarding the role of medical treatment in chronic aortic dissection.

First Author, Year [REF]	Species	Study Design	Sample Size	Medication Category	Comparison	Outcomes	Findings
Genoni, 2001 [23]	Humans	Cohort	71	Antihypertensives	Beta-blockers vs. other antihypertensive treatments	Associated complications	Favours beta-blockers
Chen, 2021 [24]	Humans	Cohort	6978	Antihypertensives	ACE inhibitors/ARBs and beta-blockers vs. other antihypertensive treatments	Mortality, associated complications	Favours ACE inhibitors/ARBs, beta-blockers
Suzuki, 2012 [25]	Humans	Cohort	503	Antihypertensives	CCBs vs. other antihypertensive treatments	Survival	Favours CCBs
Masaki, 2018 [31]	Humans	RCT	36	Lipid-lowering agents	Pitavastatin vs. control	Aortic arch growth	Favourspitavastatin
Peng, 2018 [33]	Mice, in vitro	Preclinical	40	Lipid-lowering agents	Atorvastatin vs. control (vehicle)	Autophagy, reduction of inflammation	Favours atorvastatin
Wang, 2017 [38]	Rats	Preclinical	24	Antidiabetics	Sitagliptin vs. control (vehicle)	Autophagy, moderate endothelial dysfunction	Favourssitagliptin

## Data Availability

Not applicable.
